# The BeeBiome data portal provides easy access to bee microbiome information

**DOI:** 10.1186/s12859-025-06229-7

**Published:** 2025-07-29

**Authors:** Valentine Rech de Laval, Benjamin Dainat, Philippe Engel, Marc Robinson-Rechavi

**Affiliations:** 1 Department of Ecology and Evolution, University of Lausanne, 1015 Lausanne, Switzerland; 2 SIB Swiss Institute of Bioinformatics, 1015 Lausanne, Switzerland; 3 Agroscope, Swiss Bee Research Centre, Schwarzenburgstrasse 161, 3003 Bern, Switzerland; 4 Department of Fundamental Microbiology, University of Lausanne, 1015 Lausanne, Switzerland

**Keywords:** Microbiome, Bee, Database, Metagenomics

## Abstract

Bees can be colonized by a large diversity of microbes, including beneficial gut symbionts and detrimental pathogens, with implications for bee health. Over the last few years, researchers around the world have collected a huge amount of genomic and transcriptomic data about the composition, genomic content, and gene expression of bee-associated microbial communities. While each of these datasets by itself has provided important insights, the integration of such datasets provides an unprecedented opportunity to obtain a global picture of the microbes associated with bees and their link to bee health. The challenge of such an approach is that datasets are difficult to find within large generalist repositories and are often not readily accessible, which hinders integrative analyses. Here we present a publicly-available online resource, the BeeBiome data portal (https://www.beebiome.org), which provides an overview of and easy access to currently available metagenomic datasets involving bee-associated microbes. Currently the data portal contains 33,678 Sequence Read Archive (SRA) experiments for 278 Apoidea hosts. We present the content and functionalities of this portal. By providing access to all bee microbiomes in a single place, with easy filtering on relevant criteria, BeeBiome will allow faster progress of applied and fundamental research on bee biology and health. It should be a useful tool for researchers, academics, funding agencies, and governments, with beneficial impacts for stakeholders.

## Background

A multitude of factors contribute to bee declines worldwide, but microbes have been identified to play a major role. From pathogens, like viruses or fungi causing severe diseases, to beneficial gut bacteria important for protection against pathogens or digestion of nutrients, microbes play a key role in bee fitness and survival. Sequencing efforts around the world have contributed to a better understanding of the genetic and functional diversity of microbes associated with bees [1]. These datasets are publicly available from dedicated databases, and notably include amplicon sequences, isolated genomes, shotgun metagenomes, and transcriptomes. The rapid accumulation of such datasets offers new opportunities for data integration and cross-study analyses [2]. However, such approaches are hampered by the lack of standardization in dataset annotation which makes it difficult to systematically (and automatically) search for all sequence resources of one data type.

There is agreement in the community that a centralized bioinformatics tool which would systematically catalog and provide access to sequence datasets from bee-associated microbes would be a useful resource for both fundamental and applied research [1]. One way how this can be achieved is via a data portal. A data portal is an online platform which provides access to data, not by storing the data directly, but by systematically cataloging and linking datasets deposited elsewhere. Many large-scale, data-driven projects in biology have dedicated portals, for example the TARA Oceans data portal [3]. There are also portals which provide access to data unified by a common theme, even though they were generated by multiple projects with no prior coordination. For example HumanMetagenomeDB [4] provides access to public human metagenomes, by organizing the relevant metadata. As raw data is deposited in the open databases of NCBI, EBI, and DDBJ [5–7], access to metadata linked to that raw data can be sufficient to empower users.

Here, we present the BeeBiome data portal, which automatically collects and systematically stores metadata of publicly available DNA and RNA sequence datasets of bee microbiome projects and hence makes them readily accessible to the growing research community working on bee-associated microbes. This portal will facilitate data integration and cross-study analyses with the ultimate goal to understand the ecology and evolution of bee-associated microbes and viruses, and advance our understanding of their impact on bees and bee health, from managed honey bees to solitary wild bees.

## Construction and content

### Content

The BeeBiome portal integrates metadata from all NCBI [7] available genomic or transcriptomic microbiome data for which the host is identified as Apoidea. Thanks to INSDC data sharing, this also includes all data submitted to ENA [8] or DDBJ [5]. As of 5 January 2025, BeeBiome contains 30,427 BioSamples (unique entries), encompassing 453 Bioprojects and 33,678 SRA experiments (Table 1). This represents data from 278 Apoidea host species, which includes honey bees, bumble bees, stingless bees, sweat bees, and carpenter bees (among others). All data is automatically updated every month (see Data and methods). This offers the advantage that newly deposited datasets are integrated into the BeeBiome portal on a regular basis. However, datasets with ambiguous taxonomic annotations can be missing. For example, NCBI Biosamples which are only annotated as “Bombus” without species identification are not included in the BeeBiome portal, because “Bombus” refers both to a genus and to a subgenus and hence matches two NCBI TaxIDs: 28641 (genus) and 144708 (subgenus). Such ambiguities need to be avoided to begin with, or corrected at the level of the database to have these samples integrated into the BeeBiome portal.

**Table 1:** High level BeeBiome content

GSC MIxS or NCBI package name	BioSamples
Metagenome or environmental; version 1.0	12,016
MIMARKS: survey, host-associated; version 6.0	7947
MIMS: metagenome/environmental, host-associated; version 6.0	4181
MIMARKS: survey, air; version 6.0	2351
Microbe; version 1.0	1171
Virus; version 1.0	867
MIMARKS: specimen, host-associated; version 6.0	745
MIMARKS: survey, plant-associated; version 6.0	281
MIMS: metagenome/environmental, air; version 6.0	182
MIGS: cultured bacteria/archaea, host-associated; version 6.0	179
Invertebrate; version 1.0	166
Pathogen: clinical or host-associated; version 1.0	92
MIMS: metagenome/environmental, plant-associated; version 6.0	53
MIMS: metagenome/environmental, agriculture; version 6.0	45
MIMARKS: survey, microbial; version 6.0	33
MIGS: cultured bacteria/archaea; version 6.0	32
MIUVIG: uncultivated virus genome, host-associated; version 6.0	29
MIMARKS: specimen; version 6.0	15
Pathogen: environmental/food/other; version 1.0	14
Generic	13
MIGS: eukaryote, host-associated; version 6.0	11
MIGS: cultured bacteria/archaea, agriculture; version 6.0	1
MIGS: cultured bacteria/archaea, human-associated; version 6.0	1
MIGS: cultured bacteria/archaea, miscellaneous; version 6.0	1
MIMS: metagenome/environmental, miscellaneous; version 6.0	1

For each sample, BeeBiome collects metadata which facilitates searching for relevant datasets by keywords in different categories such as ‘Organism’ (e.g., *Snodgrassella alvi*) ‘Host’ (e.g. *Apis mellifera*), ‘Library strategies’ (e.g. amplicon or WGS), ‘Library sources’ (e.g. metagenomic), or ‘Collection locality’ and ‘Collection date’. For example, to identify all amplicon sequence datasets, a user would search for Library source ‘Genomic’ and Library strategy ‘Amplicon’. Of note, it is not yet possible to automatically filter the gene amplified, for example to search only 16S rRNA gene amplicons; this information is rarely available. Filtered and sorted data can then be recovered from primary databases through BioProject, BioSample, SRA experiment, or NCBI Nucleotide identifiers, which are all linked back to the source databases. We also store assay type, center name and instrument used (e.g. “Illumina HiSeq 2000”), to allow filtering when relevant.

### Access

**Fig. 1:**
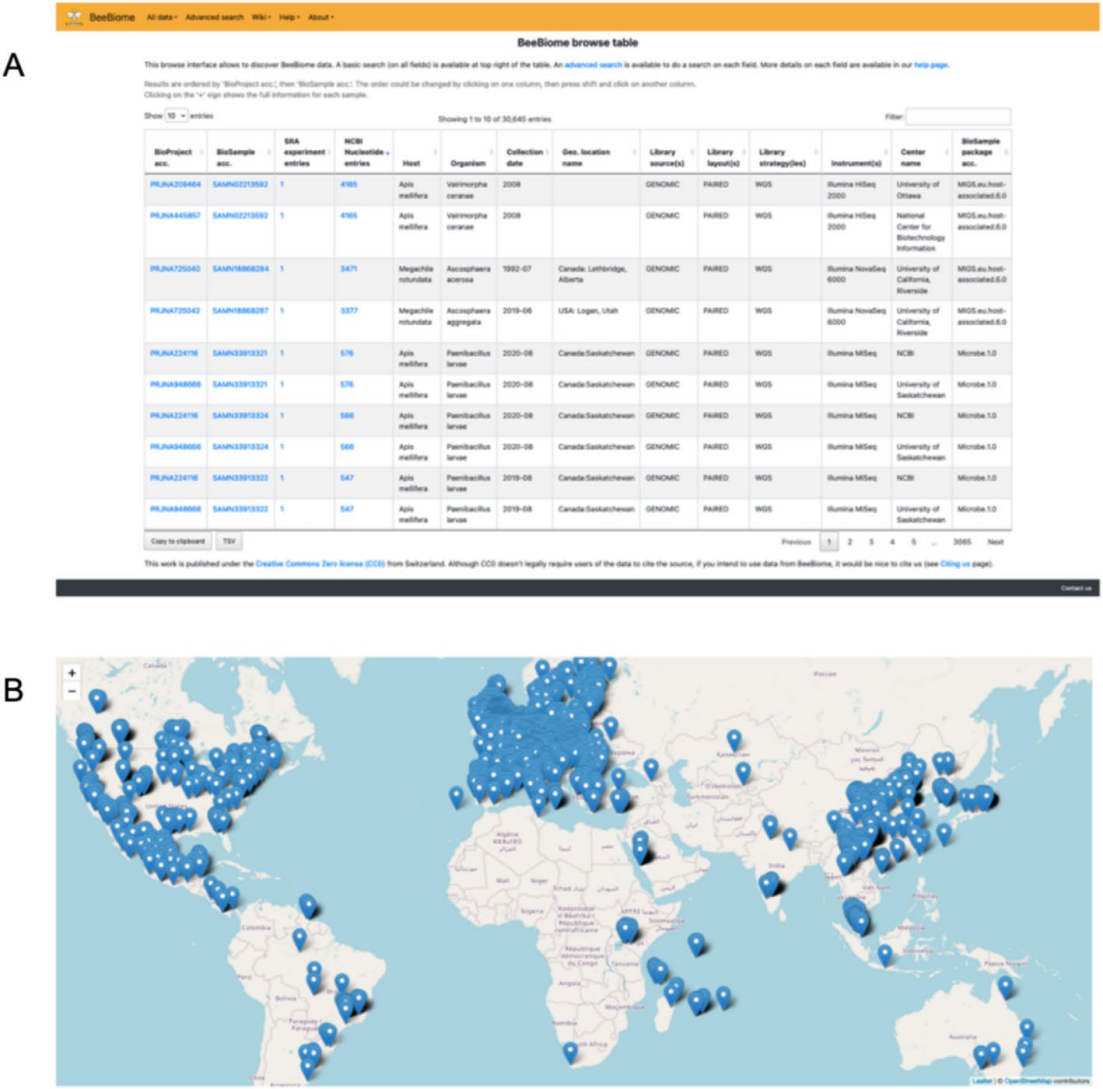
Main views of BeeBiome data. **A** “Browse Table” view; **B** Map view

The primary access to BeeBiome data is through our webpage, at www.beebiome.org. The homepage provides direct access to a ‘basic search’, as well as menus to navigate towards an ‘advanced search’, a map, and a wiki. The map simply shows the geographical location of collection for all samples for which this information is available, while ‘Browse table’ allows to see the complete table of all data in BeeBiome (Fig. 1).

**Fig. 2:**
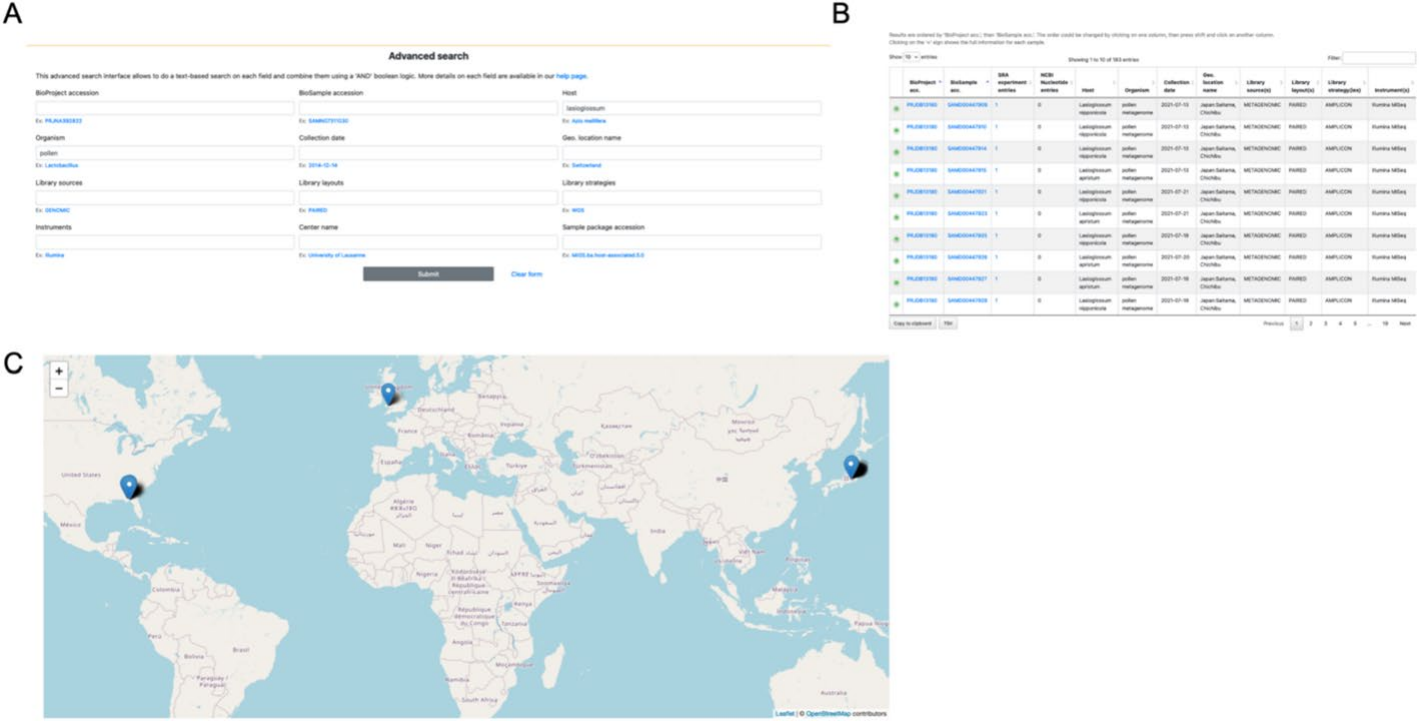
Example of advanced search in BeeBiome. **A** Search for pollen metagenomes in host Lasioglossum; **B** table of results; **C** map of results

The ‘basic search’ is a query on all metadata. Thus a query for ‘Lactobacillus’ will return 511 entries (as of 5 January 2025). The ‘advanced search’ allows querying by specific metadata fields, joined by ‘and’ (Fig. 2). There is also an option to ‘browse’ all metadata. All primary views are tables which can be filtered by terms; filtering the browse view reproduces the same results as a basic search. All tables can also be sorted by clicking on column names. Which metadata is shown adapts dynamically to the window size, while all other metadata remains accessible by unfolding each row. Whatever is shown, all metadata is used for filtering by terms. The order of metadata columns was established following a poll of the bee microbiome community, to make sure that the relevant information is visible even when window size is limited. As of writing, the first columns are thus: BioProject accession, BioSample accession, SRA experiment entries, NCBI Nucleotide entries, Host, and Organism. It should be noted that for the microbiome of e.g. the honey bee, *Apis mellifera* is the Host, while the Organism is the microbe or pest (e.g. *Snodgrassella alvi*) or the type of microbial community (e.g. insect gut metagenome). All search or filtering results, as well as the complete contents of BeeBiome, can be downloaded in TSV or copied to clipboard for easy re-use. For example, the results of a query can be downloaded as a TSV and imported to a spreadsheet software such as MS Excel, from which the Biosample IDs can be copied then used to query NCBI simply by pasting them into the NCBI search. Then they can be simply batch downloaded from e.g. NCBI SRA. The results of advanced search can also be shown on the map (Fig. 2C).

The BeeBiome wiki currently contains a comprehensive catalog of Apis and non-Apis diseases and microbes, providing an overview of most of the microorganisms known to date [1]: Apis bee diseases, including known hosts and known effects on hosts; non-Apis bee diseases, including known hosts and known effects on hosts; Apis bee microbes; and non-Apis bee microorganisms.

## Data and methods

### Data

BeeBiome stores metadata on bee microbiomes which come from NCBI Biosample, Bioproject, and SRA entries [7]. Data is retrieved using a Perl script generated by Ebot [9], modified to retrieve relevant metadata. We do not restrict metadata to those which follow a specific standard, such as GSC MIxS [10, 11] or FAANG metadata standards [12]. However, NCBI recommends submitting data according to GSC MIxS packages. These packages include attributes defined by the GSC to formally describe and standardize sample metadata. NCBI submission asks the use either of the GSC MIxS packages or of NCBI packages, forcing the submitter to give a minimum of information. Entries in BeeBiome are represented by the fields detailed in Table 2.

**Table 2:** BeeBiome entries and corresponding standards

BeeBiome entry fields	Standards
BioProject acc	PRJD# or PRJEB or PRJNA# (NCBI BioProject accession)
BioSample acc	SAMN# (NCBI BioSample accession)
SRA experiment entries	Integer
NCBI Nucleotide entries	Integer
Assay types	NCBI Strategy enum
Center name	Free text
Library layouts	SINGLE or PAIRED
Library sources	NCBI list, subset to: GENOMIC, TRANSCRIPTOMIC, METAGENOMIC, VIRAL RNA, or OTHER
Organism	NCBI Taxonomy scientific name
Host	NCBI Taxonomy scientific name
Instrument	NCBI Instrument enum
Geo. loc. name	Free text
Collection date	YYYY-MM-DD or YYYY-MM or YYYY

**Fig. 3:**
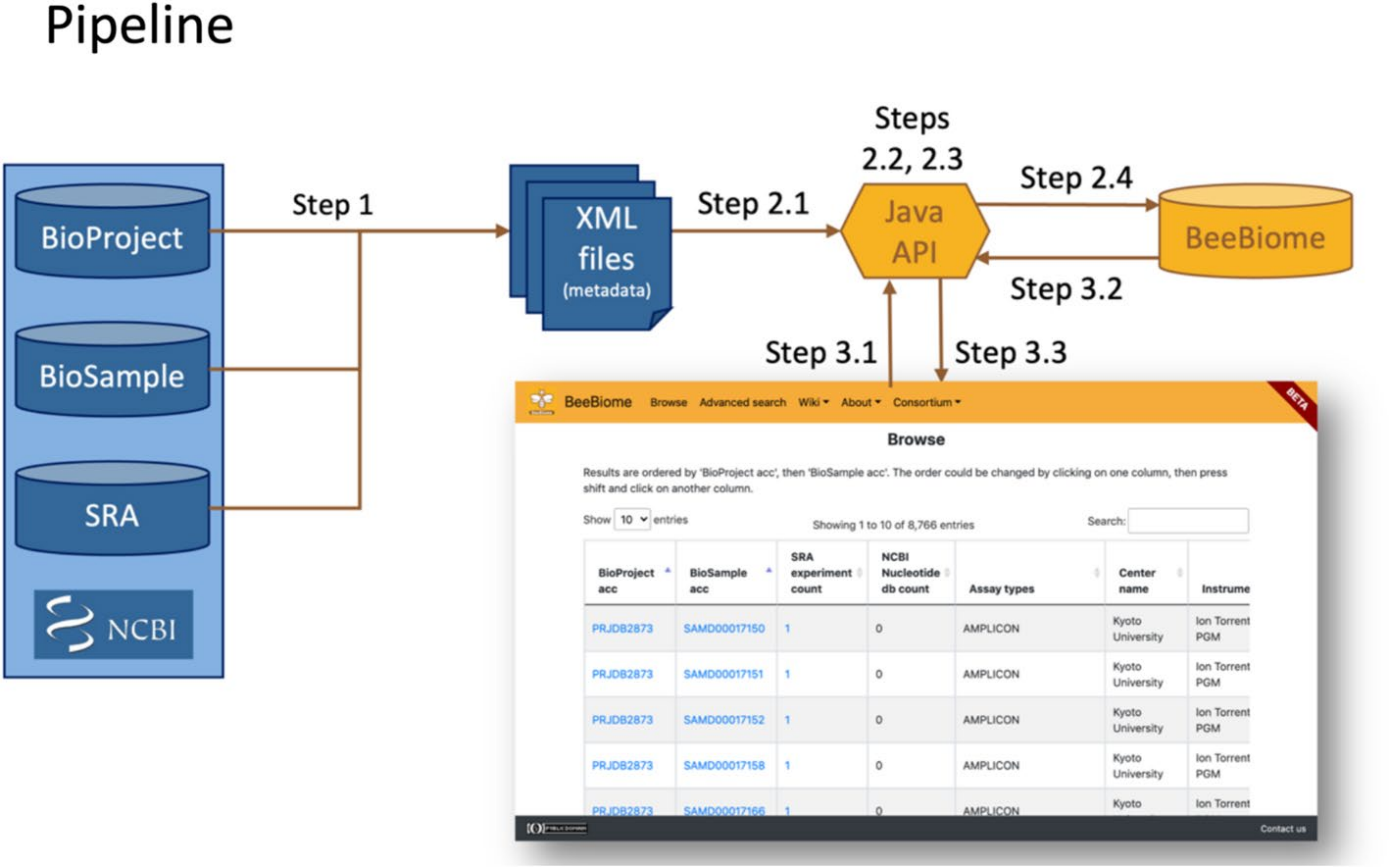
Overview of the BeeBiome database generation. Step 1: Perl script; Step 2: Java API; Step 3: Java API and React webapp

Figure 3 presents a broad overview of BeeBiome generation. The details of Step 1, to retrieve metadata, are as follows:

1. Request NCBI Taxonomy: retrieve species under the taxonomic level ‘Apoidea’. We use scientific names, common names, GenBank common names and synonyms for the next point (called *all_names*).
2. Request to NCBI BioSample using request 1 result: retrieve samples having one of the *all_names* (any fields), having an attribute named host and having an organism that is not a Metazoa or Viridiplantae.
  host [Attribute Name] AND (Apis mellifera OR honey bee OR European honey bee OR Western honey bee OR bee OR honeybee OR…) NOT Metazoa [Organism] NOT Viridiplantae [Organism] A query is built on the template ‘host [Attribute Name] AND (<names>) NOT Metazoa [Organism] NOT Viridiplantae [Organism]’, where <names> is names contained in *all_names* separated by ‘OR’.
  To avoid network and technical problems due to large files, we do several requests by generating <names_separated_by_OR> with a maximum of 300 names. Thus the same data can be recovered in different files (for instance, a BioSample can be retrieved several times).
3. Request to NCBI BioProject, NCBI SRA and NCBI Nucleotide using request 2 result: retrieve more metadata and/or links between Biosample and these databases.

BeeBiome is automatically updated every first Saturday of the month, according to these steps.

### Database and views

BeeBiome data is stored in a PostgreSQL database. The API is in Java and Spring boot, and the webapp in React. The API is used to import data from NCBI XML files:

1. Read files to put them into NCBI Java objects (built from NCBI XSD files)
2. Filter out BioSamples where the host is not one of the *all_names* (value of the attribute host–which is a free text in NCBI submit format– should be an exact match with one of the *all_names*)
3. Convert NCBI Java objects to BeeBiome Java objects (each BeeBiome Java object is equals to a table into the database)
4. Save data into database
5. An SQL view is generated to save time when there is a request. To generate this view, the query filters out biosamples with any SRA experiment.

The same API which is used to generated these views also allows to retrieve metadata in JSON format with the following URLs:

- https://beebiome.org/beebiome/sample/all for ‘Browse’ page, the ‘basic search’ restriction is done by the webapp.
- https://beebiome.org/beebiome/sample/{query} for ‘Advanced search’ page to retrieve entries with a BioSample accession containing {query}

## Utility and discussion

While generalist microbiome databases can be very powerful [13], the volume and diversity of data can be daunting, and make it difficult for small teams and researchers from diverse backgrounds to find what they need [4]. Thus we also need dedicated database portals to organize and access relevant metadata, and allow researchers to easily find datasets of interest. For example, HumanMetagenomeDB and GMrepo provide access to standardized metadata for human metagenomes [4, 14], and TerrestrialMetagenomeDB to terrestrial metagenomes [15]. Unlike BeeBiome, these databases also include manual curation, e.g. GMrepo is based on “extensive meta-data curation” [14]. Manual curation poses problems of sustainability for a small community such as bee microbiome researchers, while our automated filtering according to criteria defined by the community allows to keep BeeBiome updated continuously. Importantly, these criteria can easily be adapted or updated according to community needs and feedback. For example, following the discovery by users that our criteria could include bee associated beetle pests, we updated these criteria to exclude all of Metazoa and Viridiplantae, thus restricting to bacterial and eukaryotic microbes. To avoid manual curation, ensure metadata standardization, and enable the BeeBiome portal to correctly detect as much data as possible, an important future goal of the community should be to establish guidelines for how to deposit BeeBiome datasets into public repositories (e.g. ENA EBI checklist). This would also allow more fine-grained filtering of the datasets. For example, datasets coming from different life stages or body sites of the same host species should ideally be distinguishable. Also, while the possibility to filter datasets by library source or library strategy allows to download datasets of different types, amplicon sequence data e.g. will include datasets of amplicons coming from different genes (e.g. rpoB or 16S rRNA gene) or different regions of a given gene.

Another possible area to explore in the future is to provide access to processed datasets, and make analysis tools or pipelines available via the portal. This will facilitate data usage and help in standardizing analysis pipelines as much as possible. We hope that the community will find the current tool already helpful and help us to develop the portal further into the directions discussed above.

## Conclusions

BeeBiome provides an easy to use one-stop portal to access bee microbiome datasets. By providing the integrated access, combined with easy filtering on relevant criteria, we expect BeeBiome to improve applied and fundamental research on bee biology and health. It has already started being used by applied and academic researchers, and we expect it to be also useful to funding agencies and governments.

## Data Availability

All code is available at https://github.com/BeeBiome-consortium/beebiome-data-portal under GPL 3.0 license. All data in BeeBiome is distributed under CC0. Other information follows the original licenses, e.g. supplemental data from Engel et al. (1) is under CC-By-NC-SA 3.0.

## Abbreviations

API: Application programming interface
WGS: Whole genome sequencing

## Declarations

Ethics approval and consent to participate.

Not applicable.
